# Structure of the Type III Secretion Effector Protein ExoU in Complex with Its Chaperone SpcU

**DOI:** 10.1371/journal.pone.0049388

**Published:** 2012-11-14

**Authors:** Andrei S. Halavaty, Dominika Borek, Gregory H. Tyson, Jeff L. Veesenmeyer, Ludmilla Shuvalova, George Minasov, Zbyszek Otwinowski, Alan R. Hauser, Wayne F. Anderson

**Affiliations:** 1 Department of Molecular Pharmacology and Biological Chemistry, Feinberg School of Medicine, Northwestern University, Chicago, Illinois, United States of America; 2 Center for Structural Genomics of Infectious Diseases (CSGID), Chicago, Illinois, United States of America; 3 University of Texas Southwestern Medical Center, Dallas, Texas, United States of America; 4 Department of Microbiology and Immunology, Feinberg School of Medicine, Northwestern University, Chicago, Illinois, United States of America; The Scripps Research Institute and Sorrento Therapeutics, Inc., United States of America

## Abstract

Disease causing bacteria often manipulate host cells in a way that facilitates the infectious process. Many pathogenic gram-negative bacteria accomplish this by using type III secretion systems. In these complex secretion pathways, bacterial chaperones direct effector proteins to a needle-like secretion apparatus, which then delivers the effector protein into the host cell cytosol. The effector protein ExoU and its chaperone SpcU are components of the *Pseudomonas aeruginosa* type III secretion system. Secretion of ExoU has been associated with more severe infections in both humans and animal models. Here we describe the 1.92 Å X-ray structure of the ExoU–SpcU complex, a full-length type III effector in complex with its full-length cognate chaperone. Our crystallographic data allow a better understanding of the mechanism by which ExoU kills host cells and provides a foundation for future studies aimed at designing inhibitors of this potent toxin.

## Introduction

*Pseudomonas aeruginosa* is a Gram-negative bacterium that causes infections in hospitalized patients and individuals with cystic fibrosis [1], [2]. Because of its intrinsic resistance to many antibiotics and its propensity to acquire resistance during therapy, an urgent need exists for novel therapeutic approaches. The type III secretion system (T3SS) is one of the key virulence factors of *P. aeruginosa* and has been associated with worse outcomes in animal models of infection and in humans [3]. The cytotoxin ExoU, a 74-kDa broad-specificity phospholipase A2 (PLA_2_) [4], [5] and lysophospholipase [6], is the effector protein of this system that is most closely linked to severe disease [7]. Within the bacterium's cytosol, ExoU is bound by its chaperone SpcU, which is thought to facilitate interaction of ExoU with the type III secretion apparatus [8]. Injection of as few as 300 to 600 molecules of ExoU is sufficient to kill mammalian cells [5]. Since its discovery in 1996, concerted efforts have been made to fully understand the cytotoxicity mechanism of ExoU [9], [10], [11], [12], but they have been hindered by the absence of a three-dimensional (3D) structure.

Here we describe the 1.92 Å X-ray structure of full-length ExoU in complex with its cognate full-length chaperone SpcU. The structure confirms earlier predictions of how the two proteins interact [8] but also demonstrates surprising new features of the interaction. The structure defines the boundaries of the domains of ExoU responsible for chaperone-binding, PLA_2_ activity, and membrane localization and explains phenotypes observed with previous mutagenesis studies. Features of the structure suggest that conformational changes in ExoU induced by binding of SpcU have some features that are similar to those induced by binding of co-activators such as ubiquitinated SOD1 [11], [13]. Our crystallographic data allow a better understanding of the mechanism by which ExoU kills host cells and provides a foundation for future studies aimed at designing inhibitors of this potent toxin.

## Results

### Structure of the ExoU–SpcU complex

**Figure 1:**
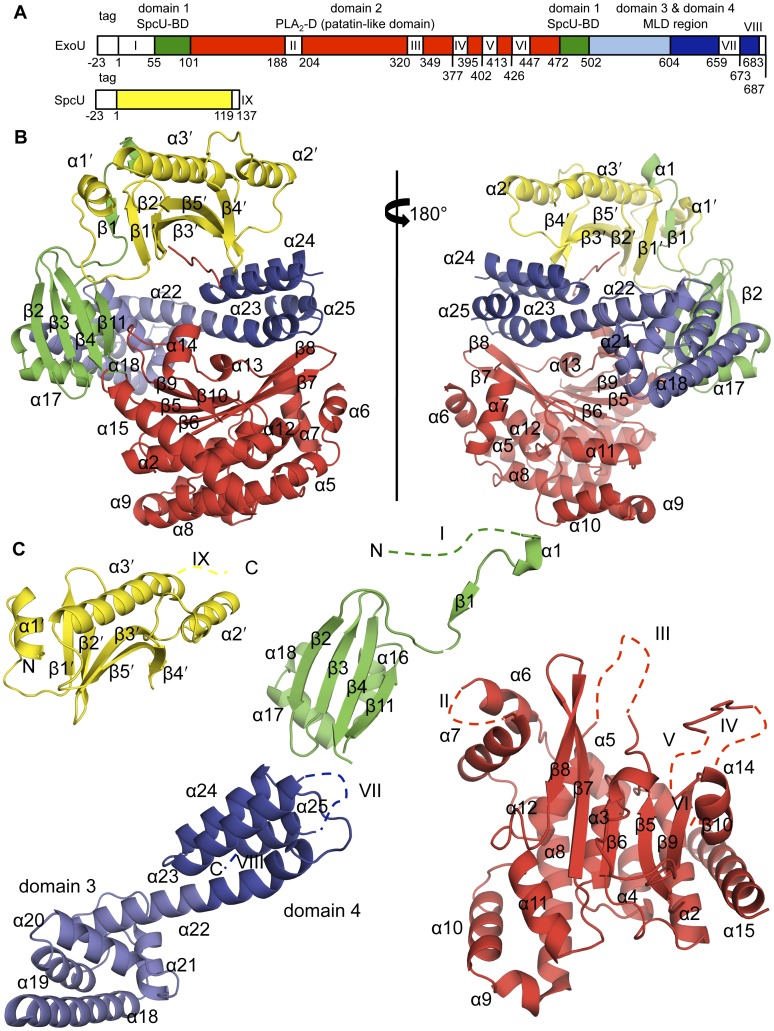
Structure of the ExoU–SpcU complex. (**A**) Constructs used for co-crystallization. Disordered regions in the structure are shown as white boxes and numbered I through IX. “Tag” refers to the N-terminal 6×His purification tag and is not numbered. (**B**) The quaternary architecture of the ∼81×67×59 Å^3^ ExoU–SpcU complex. The two proteins bury a total surface area of 2890 Å^2^. (**C**) SpcU and individual ExoU domains colored as in (**A**). The MLD consists of two separate structural regions: domain 3 (light blue) and domain 4 (dark blue). Disordered regions are shown as dashed lines and numbered as in (**A**).

The ExoU–SpcU structure is the first structure of a full-length type III effector protein in complex with its full-length chaperone. Previous structures had only full-length chaperones associated with truncated constructs of their cognate effectors [14], [15], [16], [17]. Analysis of the ExoU amino acid sequence with *DISOPRED2* [18] predicts that 25% of its sequence may be disordered and much of this is found in the protein's N-terminal region. This likely contributed to the failure of earlier attempts to crystallize the protein. Genetic and biochemical studies had indicated that SpcU, a 137 amino acid bacterial chaperone of ExoU, interacts with residues 3–123 of ExoU [8]. We therefore reasoned that the presence of SpcU might order the N-terminus of ExoU. Indeed, co-crystallization of the two proteins resulted in crystal formation and the elucidation of a 1.92 Å resolution ExoU–SpcU complex structure (Table 1). Although both proteins are full-length, approximately one-fourth of ExoU and one-eighth of SpcU remain disordered (Figure 1A). Half of the disordered ExoU residues are in the catalytic domain. SpcU provided sufficient stability to allow crystallization of ExoU, but the remaining relatively large disordered portion of the catalytic domain is consistent with ExoU being catalytically inactive in the complex with SpcU.

**Table 1:** Data collection, phasing and refinement statistics.

	Native crystal	Pt-derivatized crystal	Ta_6_Br_14_-derivatized crystal
**Data collection**			
Space group	*C*2	*C*2	*C*2
Cell dimensions			
*a*, *b*, *c* (Å)	154.1, 52.6, 119.5	154.3, 52.4, 119.9	153.9, 52.3, 119.5
*β* (°)	126.6	127.3	127.1
Resolution (Å)	30.00–1.92 (1.95–1.92)	50.00–2.50 (2.53–2.51)	30.00–3.97 (4.00–3.97)
*R*_merge_ (%)^a^	7.0 (52.0)	7.3 (N/A)	4.0 (11.0)
*I*/sigma	16.87 (2.65)	27.3 (1.4)	29.40 (10.00)
Completeness (%)	99.8 (100)	99.9 (98.2)	99.4 (81.3)
Average redundancy	3.7 (3.7)	7.3 (4.8)	4.6 (3.3)
**Refinement**			
Resolution (Å)	29.17 (1.97-1.92)		
No. of reflections	56,018 (4,088)		
*R*_work/_ *R*_free_	0.191/0.225		
No. atoms			
Protein, SpcU/ExoU	958/4,215		
Water	501		
B-factors (Å^2^)			
Protein, SpcU/ExoU	21.0/21.2		
Water	25.7		
r.m.s.d.			
Bond lengths (Å)	0.009		
Bond angles (°)	1.4		

Highest resolution shell is shown in parenthesis.

a *R*_merge_ = Σ_i_Σ_j_|*I*_ij_−<*I*_j_>|/Σ_i_Σ_j_/_ij_, where i runs over multiple observations of the same intensity and j runs over all crystallographically unique intensities. If *R*_merge_ exceeds 1.0 *Scalepack* does not report its value because it is non-informative. Instead *I*/sigma criterion is used to define resolution cut-off.

### Domain architecture of ExoU in complex with SpcU

The structure of the ExoU–SpcU complex shows how the two proteins interact and suggests that SpcU, a class IA/IB chaperone [19] (Figure S1, Table S1), defines the relative orientation of the ExoU domains. Although deletion mapping and co-transfection experiments had previously defined three functional domains within ExoU [20], [21], our structure shows that ExoU has four distinct domains defined by differences in structure and function (Figure 1A and C). Structures of other class IA/IB chaperones with peptides or truncated effector proteins had shown chaperone binding to a single effector domain. In contrast, our structure shows that SpcU interacts with three of the four domains of its cognate effector.

**Figure 2:**
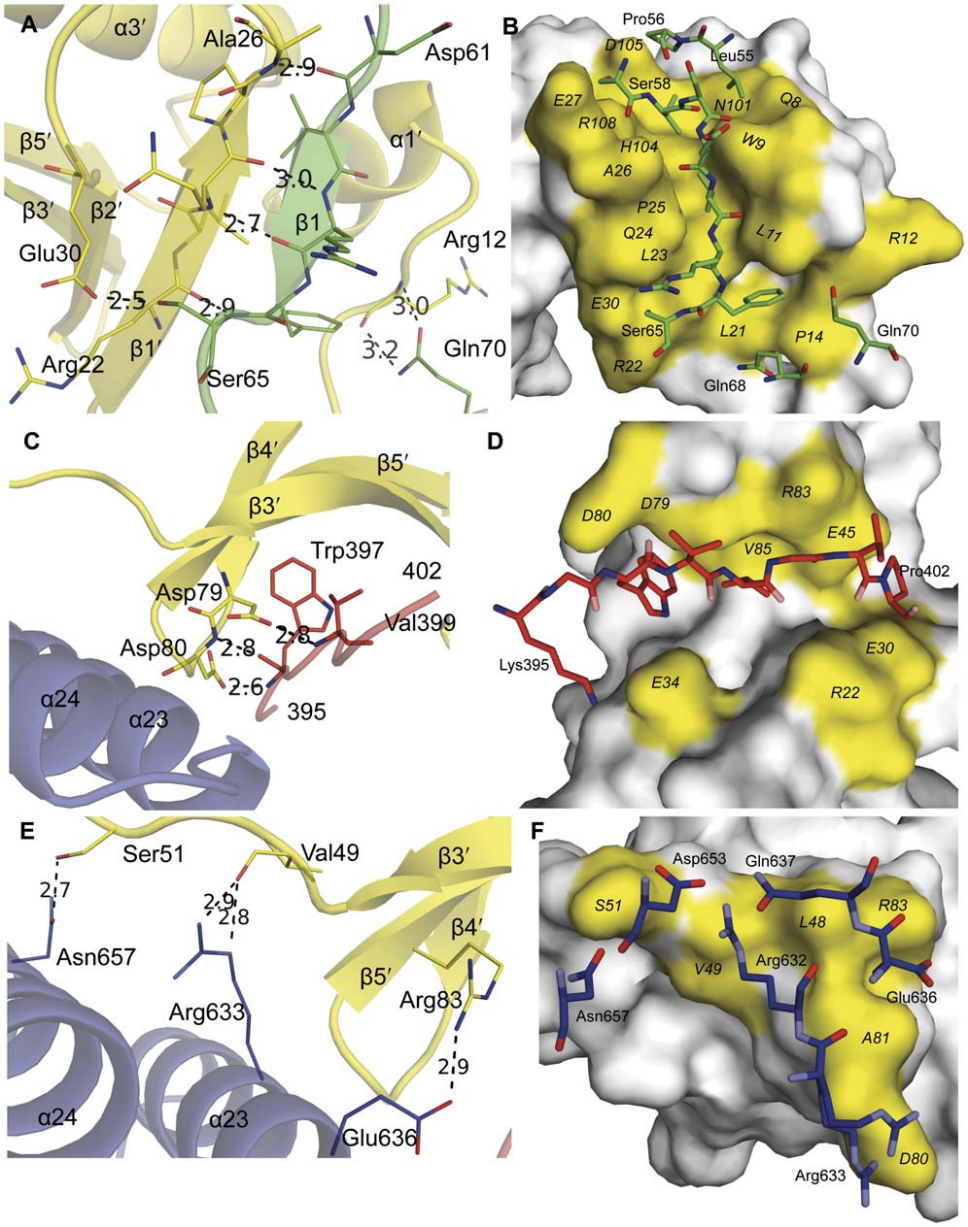
Details of the ExoU–SpcU interaction. Hydrogen bonded interactions are shown in panels A, C and E. (**A**) the SpcU-binding domain of ExoU (green); (**C**) the 395–402 region of the PLA_2_ domain of ExoU (red); and (**E**) domain 4 of ExoU (blue). SpcU is yellow in all panels. Residues involved in non-bonded interactions are shown in B, D and F. (**B**) The chaperone-binding domain of ExoU (green); (**D**) the 395–402 region of ExoU (red); and (**F**) domain 4 of ExoU (blue). In all panels, depicted peptides of ExoU and SpcU that are longer than 3 residues are labeled with the N-terminal and C-terminal residues only. Residues of SpcU in (**B**, **D**, **F**) that do not make any contacts with ExoU are in grey. Residues of ExoU are labeled in three-letter code and SpcU in one-letter code.

The chaperone-binding domain (domain 1) of ExoU is comprised of peptides from two distinct and discontinuous regions of ExoU: the previously predicted N-terminal region [8] (residues 55–101; *α*1, *β*1, *β*2, *β*3, and *β*4) that forms the most intimate interaction with SpcU (Figure 2A) and, unexpectedly, a second region (residues 472–502; *β*11, *α*16, *α*17, and *α*18) with no previously defined function (Figure 1C). The hydrogen bonds between the antiparallel *β*1′ of SpcU and the *β*1 of ExoU is a common structural motif in the binding of T3SS effectors to the class IA/IB chaperones [14], [15] (Figure 2A and B, Figure S2 and S3). Unmodeled electron density that extends from residue 55 of ExoU toward the 3_10_-helix (*α*1′) of SpcU suggests that there might be additional weak interaction surfaces. However, this region of the structure is too disordered to interpret. In spite of the poor density, it is apparent that the way those flexible residues may bind SpcU is different from the classical interpretation of a type III effector chaperone-binding domain winding around its cognate chaperone dimer.

**Figure 3:**
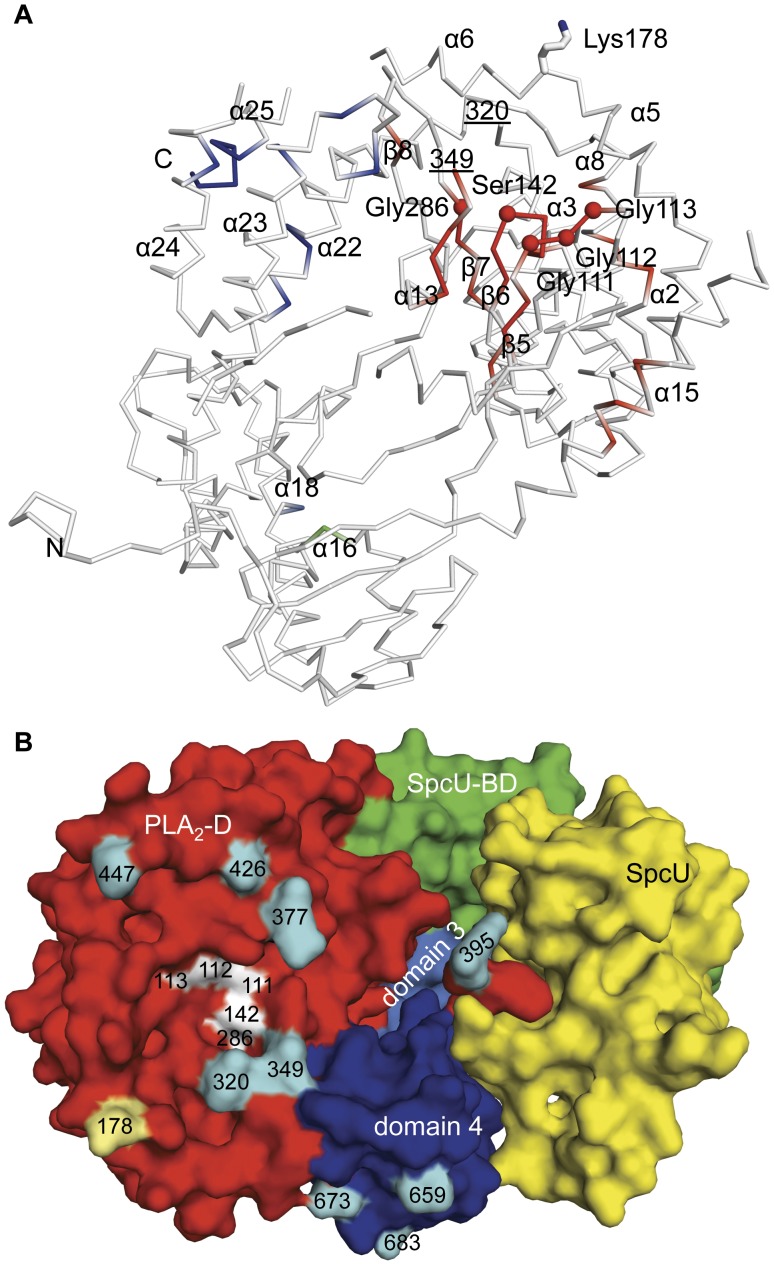
Functionally critical residues of ExoU. (**A**) Mutagenesis sites that diminish cytotoxicity of ExoU [9], [10], [21], [24], [25] (Table S3) are colored in red (the PLA_2_ domain), green (the chaperone-binding domain), light blue (domain 3) and dark blue (domain 4). The C*α* atoms of the “oxyanion hole” residues (Gly 111, Gly 112, Gly 113), the catalytic serine (Ser 142), and Gly 286 are shown as red spheres. Residues 320 and 349 mark boundaries of the disordered area containing catalytic Asp 344. Secondary structure elements that have mutation sites are labeled. The ubiquitination site of ExoU, Lys 178, is indicated. (**B**) The catalytic site residues (Gly 111, Gly 112, Gly 113, Ser 142, Gly 286 in white) of ExoU with respect to the SpcU position in the ExoU–SpcU complex. The ubiquitination site, (Lys178 in light yellow), and boundaries of disordered regions (cyan) of ExoU in the vicinity of the active site are shown. Residues 320 and 349 mark boundaries of the active site “cap” containing disordered catalytic Asp 344. Boundaries of disordered residues of domain 4 of MLD are also shown.

The PLA_2_ domain (domain 2) of ExoU had been predicted to lie between residues 107 and 357 based upon similarity with other known PLA_2_ enzymes [4], [5]. The structure of ExoU indicates that this domain starts at residue 102 but actually extends to residue 471. Amino acids after Ile 357 structurally contribute to a six-stranded antiparallel *β*-sheet, interact with a functional area known as the membrane localization domain (MLD), and bind to SpcU (Figure 1B and C, Figure 2C and D). Although the catalytic domain has relatively low sequence homology to other known phospholipases, its core *β*-sheet is structurally superimposable with those of the human cytosolic PLA_2_ [22] (cPLA_2_; Protein Data Bank (PDB) code 1CJY) and plant patatin PLA_2_ [23] (PDB code 1OXW) (Figure S4, Table S2). Some residues of ExoU previously shown to be essential for PLA_2_ activity and cytotoxicity, such as the predicted catalytic Ser 142, the “oxyanion hole” (Gly 111, Gly 112, Gly 113), and Gly 286 [4], [5], [10] are spatially adjacent in the structure, thereby explaining the previously observed phenotypes (Figure 3A and B, Table S3). The predicted catalytic Asp 344 is disordered. The structure also demonstrates that Lys 178, which is ubiquitinated in host cells [24], is solvent exposed and therefore accessible to host ubiquitin ligases (Figure 3A).

Region 550–687 had been proposed to function as the MLD [21], [24] targeting the effector protein to membranes. From the structure, however, it is clear that this region is longer, i.e. 503–687. Previous mutagenesis studies showed that relatively few insertions between residues 503 and 603 destroyed the cytotoxic activity of ExoU. In contrast, multiple insertions and substitutions between residues 604 and 687 significantly diminished or eliminated cytotoxicity [9], [10], [12], [21], [25] (Figure 3A, Table S3). Circular dichroism was used to demonstrate that several of these mutagenic alterations did not change the overall fold of ExoU [9], [24]. These results suggest that these two regions play different roles in the mechanism by which ExoU kills cells and may function as two separate structural domains, domain 3 (residues 503–603) and domain 4 (residues 604–683) (Figure 1C). These boundaries are close to those predicted by the *VAST* (Vector Alignment Search Tool) algorithm (see Materials and Methods), namely domain 3 (residues 503–618) and domain 4 (residues 619–683). The helices of domain 3 form a left-handed spiral, whereas helices of domain 4 are a helical bundle that interacts with the chaperone-binding and PLA_2_ domains. Surprisingly, domain 4 also contacts SpcU (Figure 2E and F).

### Solution studies of ExoU, SpcU and the ExoU–SpcU complex

**Figure 4:**
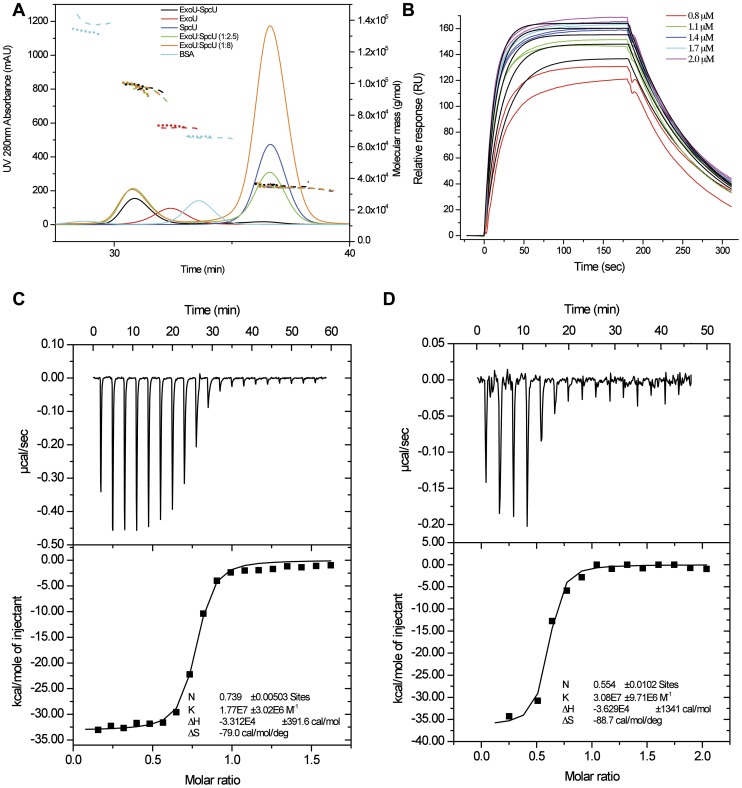
Biophysical solution studies of the ExoU–SpcU interaction. (**A**) SEC-MALS elution profiles (solid lines) of the co-expressed/co-purified ExoU–SpcU complex, ExoU, SpcU, BSA and two different molar mixtures of ExoU and SpcU using the 10 mM Tris-HCl pH 8.3 with 200 mM NaCl conditions. Molecular mass distribution at 200 mM NaCl (dotted lines) and 500 mM NaCl (dashed lines) of the samples are shown. (**B**) The SPR experimental curve-fitting methodology for a simple 1∶1 binding model for immobilized SpcU with ExoU as the analyte. Pairs of colored traces at each concentration indicate duplicate experimental determinations; black traces show the corresponding binding model curves. A 1∶2 ExoU∶SpcU model was used to characterize binding of SpcU to immobilized ExoU (not shown; see text for the rates). (**C and D, upper panels**) Injection of SpcU (120 µM – dimer concentration) into ExoU (15 µM) and ExoU (50 µM) into SpcU (5 µM – monomer concentration) respectively, produced dose-dependent, exothermic responses. The integrated data (filled black squares, lower panels in **C** and **D**) could be fitted to a single set of sites (black fitting curve). N – binding stoichiometry, *K* – binding constant related to *K_d_* by 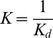, Δ*S* – entropy change; and Δ*H* – enthalpy change.

The affinity and stoichiometry of the interaction of ExoU and SpcU in solution was also investigated. The crystal structure of the ExoU–SpcU complex suggests that the SpcU dimer would bind two ExoU monomers. To investigate the oligomeric state in solution, size exclusion chromatography with multi-angle laser light scattering (SEC-MALS) was used to estimate molecular weights of ExoU, SpcU and the co-expressed ExoU–SpcU complex, all cloned as full-length tagged proteins. ExoU was ∼72–73 kDa (theoretical mass is 76.7 kDa), SpcU as a dimer was ∼34–35 kDa (theoretical mass of a monomer is 17.7 kDa) and the ExoU–SpcU complex was ∼97–99 kDa, a value consistent with either an SpcU monomer or dimer binding an ExoU monomer (theoretical mass is 94.3 kDa, or 112.0 kDa) (Figure 4A) though the variation of size across the peak suggests that the complex is undergoing dissociation and the peak is a mixture of ExoU monomers binding both SpcU dimers and monomers. The strength of the ExoU–SpcU interaction was evaluated by surface plasmon resonance (SPR) using immobilized SpcU and ExoU as an analyte at concentrations between 0.8–2.0 µM. The association and dissociation rate for ExoU was 3.56(3)×10^−4^ M^−1^s^−1^ and 0.01(1) s^−1^, respectively. The binding affinity (*K_d_*) was calculated as 306 nM (Figure 4B). Although the SPR experiment is measuring binding to immobilized protein, this should be a good estimate of solution affinity since binding of dimeric SpcU to immobilized ExoU is consistent with these values (*K_d_* = 113 nM; *k_a_* = 1.11(1)×10^−5^ M^−1^s^−1^; *k_d_* = 0.01(4) s^−1^). Nevertheless, SPR measurements can be affected by mass transport effects, excluded volume effects, surface concentration, and protein immobilization effects. The latter can affect the apparent affinity of protein-protein interactions. Since isothermal titration calorimetry (ITC) is an equilibrium-binding assay, it should be a more reliable method than SPR for calculating equilibrium dissociation constants. Therefore, we used ITC to validate our SPR data. ITC provided *K_d_* values of 57 nM (when ExoU was titrated with SpcU) and 33 nM (when SpcU was titrated with ExoU) (Figure 4C and D).

**Figure 5:**
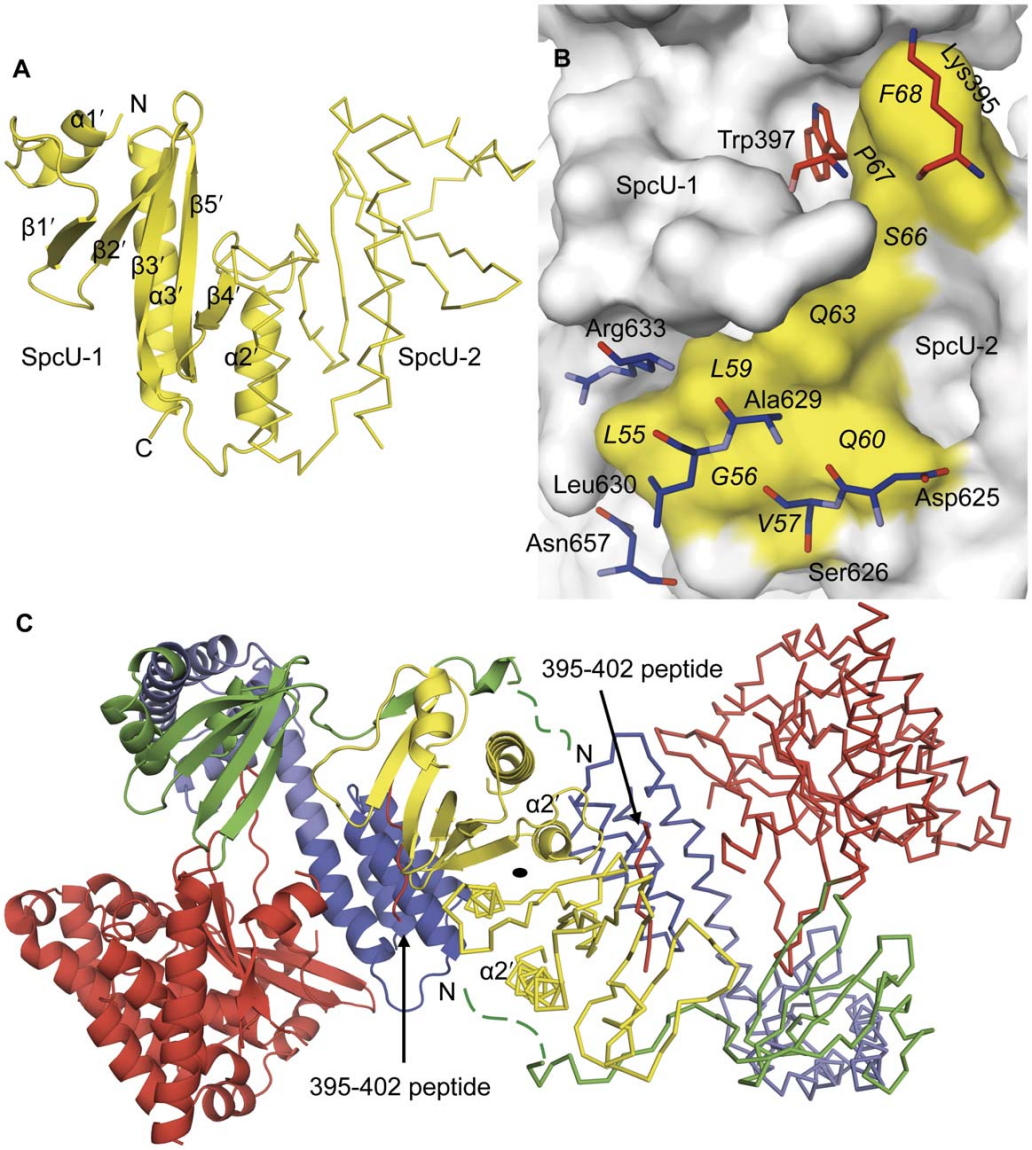
SpcU dimer and crystal packing. (**A**) SpcU-1 from the asymmetric unit forms a dimer with a symmetry-related SpcU-2. Two subunits bury a total surface area of 1870 Å^2^ at their *α*2′ helices. (**B**) Non-bonded contacts between the 395–402 region (red), domain 4 (blue) of ExoU from the asymmetric unit and the symmetry-related SpcU (SpcU-2; yellow). Residues of ExoU are labeled in three-letter code and those of SpcU in one-letter code. (**C**) The 2×(ExoU–SpcU) complex with 9352 Å^2^ in total buried surface area. The disordered N-terminal residues of ExoU are shown as dashed green lines. The 395–402 peptide of the PLA_2_ domain of ExoU is indicated with an arrow. The asymmetric unit is shown in ribbon, whereas symmetry-related molecules are depicted as the C*α* traces.

The structure of the ExoU–SpcU complex along with the SEC-MALS measurements shed light on the oligomerization state of the two proteins. SpcU in solution (Figure 4A) and in the crystal with ExoU forms a dimer (Figure 5A), which is similar to dimers of other class IA/IB chaperones [19]. The symmetry-related SpcU in the dimer also interacts with ExoU, i.e. domain 4, from the asymmetric unit (Figure 5B). While the SpcU–SpcU dimer relates two ExoU–SpcU complexes at the crystallographic two-fold axis (Figure 5C), our SEC-MALS and ITC results (Figure 4A, C and D) indicate that an ExoU monomer can bind either an SpcU monomer or dimer in solution. If the two ExoU monomers bound per SpcU dimer is indeed a crystal artifact, then the portions of the second SpcU molecule that forms an interface with the 395–402 peptide of the second ExoU molecule in the crystal may be available in solution to bind to the disordered N-terminal 54 residues of the first ExoU molecule. In other words, the N-terminal residues of a single ExoU molecule may contact two SpcU molecules, yielding a 2∶1 SpcU∶ExoU complex in solution. This type of interaction is supported by similar observations with other effector protein–chaperone complexes (Figure S2 and S3) [14], [15], [16], [17], [19], [26] and suggests that the N-terminal residues of a single ExoU molecule wind around the SpcU dimer, which acts as a spool. Subsequent displacement of these residues would make them available for targeting and/or loading into the type III secretion apparatus.

### Activation of ExoU

**Figure 6:**
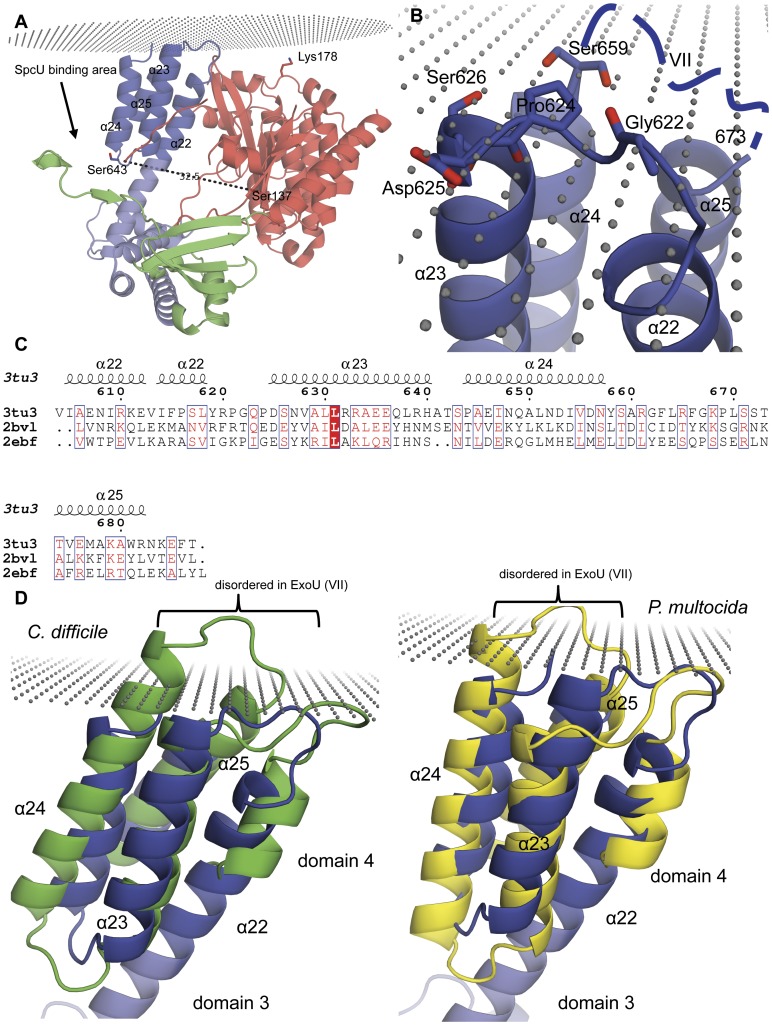
Localization of ExoU to the membrane. (**A**) A hypothetical model of an ExoU–co-activator complex associated with the membrane. It is presumed that a eukaryotic co-activator (not depicted) binds and results in the same relative orientation of the ExoU domains seen in the structure of the ExoU–SpcU complex. The distance between the C*α* atoms of Ser 137 and Ser 643 of ExoU is shown in Å and approximates that observed with ExoU bound to co-activator [11]. (**B**) Close-up view of the ExoU–membrane interaction. Residues of domain 4 that are predicted to be embedded into the membrane are shown. A loop spanning residues 660 through 672 (blue dashed line), which is disordered in the structure, may also interact with the membrane. (**C**) Multiple sequence alignment of the *Pasteurella multocida* toxin (PDB code 2EBF; N-terminal residues 590–670; yellow), *Clostridium difficile* toxin B (PDB code 2BVL; N-terminal residues 3–84; green), and ExoU (residues 604–687 of domain 4; colored as in (**A**)). Residues in the red box with white characters have strict identity; residues indicated by red characters are similar within a group; residues shown in blue frames are similar across groups (see Materials and Methods). (**D**) Structure superposition of domain 4 of ExoU with toxin B of *C. difficile* (left) and *P. multocida* toxin (right). Secondary structure elements of domain 4 of ExoU are shown. Only sequences of ExoU are numbered. Disordered residues 660–672 (region VII, see (**B**) and **Fig. 1A**) of domain 4 of ExoU are depicted. The lipid carbonyl groups in (**A**, **B**, and **D**) are shown as small grey spheres.

**Figure 7:**
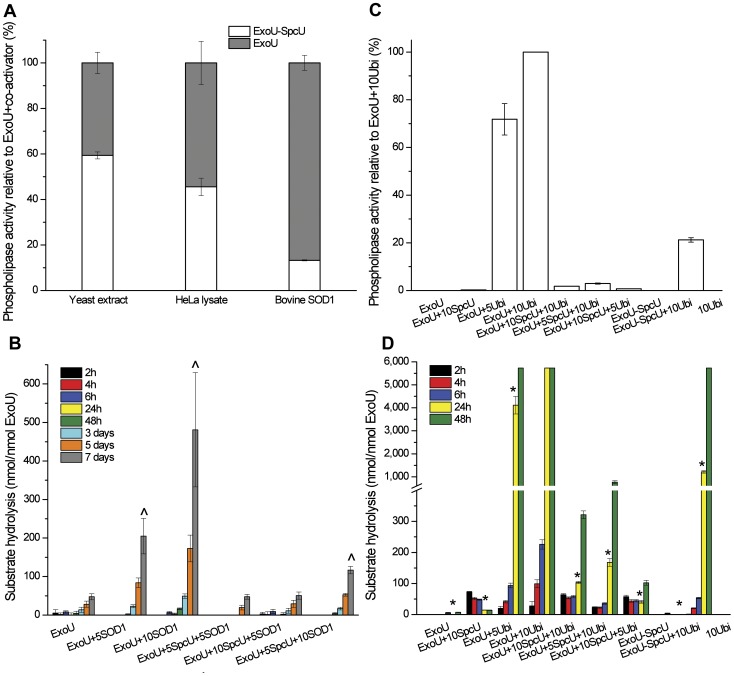
The PLA_2_ activity of ExoU. (**A**) The phospholipase activity of the ExoU–SpcU complex was compared to that of ExoU alone in the presence of different activators; results are normalized to 100% activity for ExoU plus activator in the absence of SpcU. (**B**) Activation of ExoU by 5 (5SOD1) and 10 (10SOD1) molar excess of SOD1 in the presence of 5 (5SpcU) and 10 (10SpcU) molar excess of SpcU. (**C**) Comparison of the phospholipase activity of ExoU with monoubiquitin as co-activator; results are normalized to 100% activity of ExoU with 10 molar excess of monoubiquitin (ExoU+10Ubi) at 24 hr. (**D**) Activation of ExoU with addition of 5 (5SpcU) or 10 (10SpcU) molar excess of the chaperone, and of ExoU in complex with SpcU (ExoU–SpcU) by 5 (5Ubi) or 10 (10Ubi) molar excess of monoubiquitin. Error bars show the standard errors of the mean, where n = 3. The Student's t test was used to compare measurements in PLA_2_ assays. ∧ and * are statistically different (P<0.05) from ExoU in (**B**) and ExoU+10Ubi at 24 hr in (**D**), respectively.

Ubiquitin is a known activator of ExoU [13], and our structural data suggest a possible model of ExoU and ubiquitin interaction. Benson et al. [11] used double electron-electron resonance (DEER) to show that ExoU by itself exhibited multiple conformations with a distance from Ser 137 to Ser 643 ranging between 22–38 Å. In contrast, the toxin favored a single conformation in the presence of a co-activator (presumably ubiquitinated SOD1) with this distance being 30.6±2.0 Å. Surprisingly, these two serine residues are 32.5 Å apart in our structure (Figure 6A). Although there may be other domain arrangements that result in the same distance, the simplest interpretation is that binding of ubiquitinated proteins and binding of SpcU to ExoU result in similar orientation of the toxin's domains. In addition, SpcU competed with ubiquitinated SOD1, ubiquitin and other activators (e.g. yeast extract and HeLa cell lysate), reducing the PLA_2_ activity associated with ExoU (Figure 7). The co-expressed/co-purified ExoU–SpcU complex hydrolyzed a synthetic phospholipid analog (arachidonoyl thio-phosphatidylcholine) only with the addition of eukaryotic stimulators and less efficiently than ExoU in the absence of SpcU. SpcU inhibited hydrolysis of the phospholipid analog even in the presence of excess ratios of SOD1 (Figure 7). In these assays, residual activity may be accounted for by a small amount of SpcU-free ExoU. Indeed, the SEC-MALS analysis of the co-expressed/co-purified ExoU–SpcU complex revealed traces of free SpcU dimer (Figure 4A) that may be due to its dissociation from ExoU at higher ionic strength (500 mM NaCl used in the SEC-MALS experiment and 300 mM NaCl plus 20 mM CaCl_2_ used in the PLA_2_ assay buffer). On the other hand, minor excess of free SpcU dimer may reflect its higher level of expression in *Escherichia coli*. Elution of some free SpcU dimer was also observed at 200 mM NaCl. Together, our structure and the DEER results [11] are consistent with ubiquitin and ubiquitinated proteins (e.g. SOD1) causing a similar overall ExoU configuration as SpcU. However, whereas ubiquitin and ubiquitinated proteins [13] form catalytically active complexes with ExoU, the ExoU–SpcU complex is catalytically inactive. Thus, co-activators may also cause disordered regions around the active site of the PLA_2_ domain of ExoU to become structured.

### ExoU–host membrane model

The molecular mechanisms of ExoU targeting and cleavage of substrate phospholipids within the host are still unknown. Our structure and the DEER data [11] suggest that SpcU and ubiquitinated proteins may orient the ExoU domains in a similar manner. Consequently, we used the ExoU structure from the ExoU–SpcU complex to model the ExoU–co-activator membrane interactions. Interestingly, the *PPM* server (see Materials and Methods) predicted that only residues of domain 4 of the MLD region were embedded in membrane (Figure 6A and B). This same portion of the MLD region has been previously implicated by mutagenesis studies to be necessary for membrane localization [20], [21], [24], [25]. Consistent with this interpretation, the four-helix bundle fold of domain 4 of ExoU has been reported for other bacterial toxins with known 3D structure and annotated as a membrane localization domain [27], [28], [29], [30] (Figure 6C and D). The *PPM* server predicted the four-helix bundle domain of those toxins being also embedded in membrane (not shown). Furthermore, the membrane localization domain of the Rho GTPase inactivation domain (RID) of the *Vibrio cholerae* multifunctional autoprocessing repeats-in-toxin (MARTX) toxin [31], [32] also shows sequence similarity to the membrane localization domains in Figure 6C. In the aforementioned ExoU–membrane model, the active site of ExoU was oriented toward membrane, as would be expected for a phospholipase. Thus, co-activators of ExoU may play a role analogous to the C2 domain of the related enzyme human cPLA_2_ and function to allow targeting to membranes [22], [33]. However, since this model is based upon the structure of ExoU bound to SpcU rather than a co-activator, the actual membrane interactions of ExoU within host cells may differ somewhat. Furthermore, a structure we have determined of another MLD (PDB code 4ERR; [34]) suggests that the four helix bundle can open up, possibly presenting a hydrophobic surface for membrane interactions. Consequently, one should be cautious about extrapolating membrane interactions from structures of soluble complexes.

## Discussion

### The ExoU–SpcU structure

Here we present the 1.92 Å crystal structure of the *P. aeruginosa* full-length cytotoxin ExoU in complex with its cognate full-length chaperone SpcU. Consistent with previous genetic and biophysical data our high-resolution crystal structure suggests how the two proteins interact in the bacterium's cytosol, prior to secretion. This complex may facilitate the storage of the toxin and its appropriate delivery to the type III secretion apparatus [19]. Based on our SEC-MALS and ITC data, the two proteins apparently exist in equilibrium between the 1∶1 and 1∶2 ExoU∶SpcU stoichiometries. This is consistent with our crystallographic data and the native mass spectrometry and structural results of Gendrin et al. [35]. The unusual ExoU∶SpcU 2∶2 ratio shown in Figure 5C has been reported between the *P. aeruginosa* anti-activator ExsD(1–46) construct and its ExsC chaperone, and between the *Yersinia pestis* truncated regulatory protein YscM2(1–56) construct and its SycH chaperone [36], [37]. Both constructs lack a C-terminal part and are, therefore, too short to wind around a chaperone dimer, i.e. to interact with another hydrophobic patch of a chaperone located underneath its *β*-sheet and to make additional strand-strand interaction. Thus, two molecules of ExsD and YscM bind identical sites on each of the corresponding chaperone molecule in a dimer resulting in a 2∶2 complex. The question whether the ExoU–SpcU interaction in the crystal is the same in solution especially with the residues Lys 395–Pro 402 binding the aforementioned hydrophobic patch of SpcU and the first 54 N-terminal residues of ExoU remaining disordered, remains a matter of a discussion. Furthermore, our structure shows that domain 4 of the membrane localization region of ExoU also binds SpcU and, thus, it makes our structure the first example of a class IA chaperone interacting with three functionally and structurally different domains of a type III effector protein.

Our PLA_2_ assays of ExoU in the co-expressed/co-purified complex with SpcU or with added SpcU demonstrated that the chaperone significantly reduces phospholipase activity towards the synthetic substrate arachidonoyl thio-phosphatidylcholine in the presence of eukaryotic co-activators. Despite the published higher affinity of ExoU for monoubiquitin (*K_d_*∼1.4 nM) [13] compared to our measured affinity for SpcU (tens to hundreds of nM by ITC and SPR, respectively), SpcU can block activation of ExoU when added separately or when present in the co-purified ExoU–SpcU complex. A structural model of an ExoU–co-activator complex is not available, but the DEER results [11] and our structure suggest that both SpcU and a co-activator (e.g. ubiquitin or ubiquitinated protein) may result in a similar relative orientation of the ExoU domains. Whether SpcU and a co-activator have similar binding sites on ExoU remains unclear, but a co-activator must induce additional local structural rearrangements in ExoU (especially in the catalytic phospholipase domain) not seen in the present structure that trigger PLA_2_ activity.

### Comparison of the ExoU–SpcU complex structures

While our manuscript was under review, a 2.94 Å resolution structure between the truncated constructs, ExoU(30–687) and SpcU(1–127), from *P. aeruginosa* (PDB code 4AKX) was determined by the single-wavelength anomalous dispersion (SAD) method and published by Gendrin et al. [35]. These results were interpreted to indicate that ExoU has three distinct domains and that SpcU interacts only with the N-terminus of ExoU (residues 28–65). In contrast, our findings indicate that ExoU has four distinct domains and that SpcU interacts with the N-terminus, the PLA_2_ domain and domain 4 of the membrane localization region of ExoU. Because of these different interpretations, we compared the two structures.

The two structures should be very similar because they have the same space group and similar unit cell parameters. Indeed, the root-mean-square deviation (r.m.s.d.) values were 0.4 Å over 119 C*α* atoms of SpcU and 1.0 Å over 500 C*α* atoms of ExoU. Similar disordered regions were also observed in both structures. Although approximately one-fourth of the ExoU sequence was not built in either structure, we observed some residual density of those regions implying that they are disordered and not absent due to protein degradation and/or proteolysis, though that is not completely excluded in either structure. However, closer examination of the 4AKX structure with the available structure factors revealed three significant structural discrepancies.

**Figure 8:**
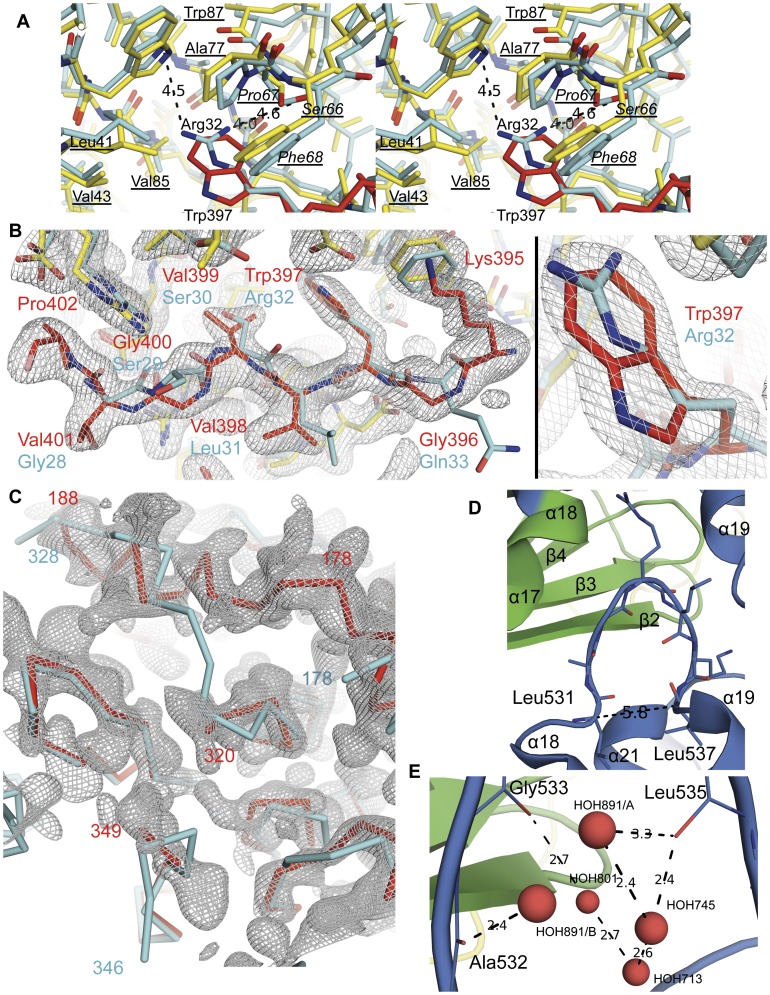
Comparison of the ExoU–SpcU structures. (**A**) A stereo-view of the superposed 3TU3 and 4AKX (cyan) structures showing differences in the ExoU models. (**B**) Residues of the 395–402 region of the PLA_2_ domain (carbon atoms are in red) and SpcU (carbon atoms are in yellow; asymmetric unit residues are underlined; symmetry-related residues are underlined italic) of 3TU3 are shown. Distances from the guanidinium group's nitrogen atoms of Arg 32 of ExoU to the N*ε*1 atom of Trp 87, the O*γ* atom of Ala 78 of SpcU from the asymmetric unit and the O*γ* atom of Ser 66 from a symmetry-related SpcU in 4AKX (carbon atoms are in cyan) are shown. The 1*σ* 2*F*_o_ – *F*_c_ electron density map of the 3TU3 structure is displayed. (**C**) The 3TU3's 1*σ* 2*F*_o_ – *F*_c_ electron density map with the C*α* traces of the superposed 3TU3 (red) and 4AKX (cyan) structures in the area of the active site “cap”. (**D**) The *Ω*-loop (residues 531–537) of domain 3 of ExoU of 3TU3 that are disordered in the 4AKX structure. The loop is positioned between SpcU-binding domain of one symmetry-related ExoU and domain 3 of another symmetry-related ExoU in 3TU3. (**E**) Water molecules stabilize the *Ω*-loop conformation.

One difference is that our structure shows Lys 395–Pro 402 of the PLA_2_ domain interacting with a hydrophobic patch underneath the β-sheet of SpcU. Gendrin et al. interpreted this density as being residues Gly 28–Gln 33. They introduced a thrombin site between residues 29 and 30 of ExoU that should result in an ExoU(30–687) construct with Gly 28 and Ser 29 of the amino acid sequence reported in the PDB being residues of the thrombin site. However, the presence of a strong positive difference density peak and clear density in the 2*F*_o_ – *F*_c_ map before the N atom of Gly 28 suggests continuation of the peptide chain, i.e. the presence of a non-reported residue 27. In our model this electron density corresponds to residues Lys 395–Pro 402 of the PLA_2_ domain and the aforementioned positive peak is where the Pro 402 is located (Figure 8A and B). A major problem with the Gly 28–Gln 33 interpretation is that they have Arg 32 aligned with Trp 397 in our structure. The side chain of Arg 32, i.e. its guanidinium group, is embedded in a hydrophobic pocket created by the side chains of Leu 41, Val 43, Ala 77, Val 85 and Trp 87 of SpcU from the asymmetric unit and the side chains of Pro 67 and Phe 68 of a symmetry-related SpcU (Figure 8A). Although the aliphatic part of arginine side chain may be positioned near adjacent hydrophobic residues in proteins [38], its hydrophilic guanidinium group would be expected to be facing solvent or charged groups on other amino acids. Moreover, there was also a positive difference density peak in the 4AKX map at the position where we placed the N*ε*1 atom of Trp 397. This feature disappeared after one cycle of refinement against their data when Arg 32 was mutated to tryptophan in 4AKX. Additionally, the position of Val 399 in 3TU3 instead of the modeled Ser 30 in 4AKX makes sense given the interaction of ExoU with the hydrophobic patch of SpcU (Figure 8B). An additional piece of evidence for the correctness of our model comes from our experimental electron density maps, showing that residues preceding Leu 55 do not wind around the SpcU dimer, but pack between SpcU and the PLA_2_ domain of a symmetry-related ExoU (not shown). Thus, although modeling of residues Gly 28–Gln 33 in 4AKX are consistent with similarities in the chaperone–effector interactions reported in the literature, they are not supported by the low-resolution experimental phases.

Second, because of the low resolution and structural resemblance of the PLA_2_ domain of ExoU with the human cPLA_2_, residues Pro 320–Leu 328 were interpreted as ordered and modeled in 4AKX by Gendrin et al.. Based on the quality of the 4AKX electron density map we think that this region should be considered as disordered in 4AKX. Pro 320–Leu 328 are part of an active site “cap” – a flexible region that also contains the catalytic Asp 344 that is not seen in either structure. On the other hand, residues Lys 179–Ser 188 in 3TU3 have a clear continuous electron density that conflicts with the interpretation of residues 320–328 in 4AKX (Figure 8C). Residues 179–188 are not present in 4AKX. However, a strong positive difference density peak in the 4AKX electron density map is aligned with the C*α* atom of Lys 178 of the superposed 3TU3 structure. Examining lower electron density levels in the 2*F*_o_ – *F*_c_ density map for 4AKX reveals electron density similar to that for Lys 179–Ser 188 in 3TU3, whereas no density appeared between Pro 320 and Phe 322 in 4AKX. We agree with Gendrin et al. that the “cap” region and neighboring residues Lys 179–Ser 188 may be flexible and may change their conformation to accommodate a substrate. For example, while ordered, the Lys 179–Ser 188 peptide in our structure exhibits higher B-factors than, for example, residues preceding Lys 178. However, the high-resolution phases indicate that the position of the Lys 179–Ser 188 region in 3TU3 is correct and residues Pro 320–Leu 328 in 4AKX appear to be a model error.

Third, residues Leu 531–Leu 537 in 3TU3 represent a structure element, known as an omega-loop, that is commonly found on a protein's surface and may be important for function and stability [39], [40]. The loop is stabilized by interactions with the SpcU-binding domain of one symmetry-related ExoU and domain 3 of another symmetry-related ExoU. (Figure 8D). There are also water molecules that stabilize the loop conformation in 3TU3 (Figure 8E). This loop is not modeled in 4AKX.

## Conclusion

Our structure of the ExoU–SpcU complex indicates a role for the chaperone in defining the relative orientation of the ExoU domains, demonstrates new features of effector protein–chaperone interactions, allows interpretation of previous genetic and biophysical data, and provides an important tool for further understanding of the mechanism by which ExoU kills host cells.

## Materials and Methods

### Cloning, expression, and purification

The full-length *P. aeruginosa exoU* and *spcU* genes were cloned in the pMCSG7 vector utilizing a ligation independent cloning protocol and transformed into BL21magic *E. coli* cells for expression, as previously described [41]. For co-expression of ExoU with SpcU, the toxin was re-cloned in the pMCSG21 vector to select for a different resistance marker, spectinomycin. The cells were grown at 37°C to OD_600_ = 1.0, cooled to 16°C, and induced with 1 mM isopropyl-1-thio-D-galactopyranoside. After overnight incubation, cells were spun down, resuspended in buffer A containing 10 mM Tris–HCl pH 8.3, 500 mM NaCl, 5 mM 2-Mercaptoethanol and lysed by sonication. The 6×His-tagged ExoU and SpcU were eluted from a Ni-NTA column (GE Healthcare, Piscataway, NJ) in buffer A plus 500 mM imidazole. The collected fractions were further purified on a Superdex 200 gel filtration column (GE Healthcare, Piscataway, NJ) using buffer A. The homogeneity of the proteins was analyzed by SDS-PAGE and dynamic light scattering.

### PLA_2_ assay

Catalytic activities of ExoU were assessed using the cPLA_2_ assay approach (Cayman Chemicals, Ann Arbor, MI), as previously described [9]. First, HeLa cell lysates were generated by washing 10-cm plates of HeLa cells (80% confluence) three times with cold PBS (Mediatech, Inc., Manassas, VA). Cells were collected into 1 ml cold PBS using a cell scraper (Sarstedt, Newton, NC) and lysed by repeated passage through a 27-gauge needle. Lysates were then cleared by ultracentrifugation at 100,000× *g* at 4°C for 1 hr. Yeast cell lysates from *Saccharomyces cerevisiae* were prepared by lysis with Y-PER Yeast Protein Extraction Reagent (Pierce, Rockford, IL), cleared by centrifugation at 18,000× *g* at 4°C for 5 min. Purified bovine liver-derived superoxide dismutase 1 (SOD1) (Sigma-Aldrich Corp., St. Louis, MO) and bovine monoubiquitin (Sigma-Aldrich Corp., St. Louis, MO) were also used for this assay. A total of 5 µg of purified ExoU (65 pmol) or 5 µg of the co-purified ExoU–SpcU complex (53 pmol ExoU) were incubated with 200 µL of the assay substrate (1.5 mM arachidonoyl thio-phosphatidylcholine) and 15 µL of a precleared HeLa cell lysate, yeast cell lysate, or bovine SOD1 at room temperature. The activity was quantified by measuring the *A*_405_ at various time points after addition of 10 µL of 25 mM 5,5′-dithiobis(2-dinitrobenzoic acid) (DTNB). The PLA_2_ activity of ExoU was calculated using the following formula: *A*_405_/10.00(extinction coefficient for DTNB)×1/(amount ExoU [nmol]). For PLA_2_ assays of ExoU with free SpcU (not co-purified with ExoU), the same amount of ExoU was used (65 pmol). SOD1, monoubiquitin, and SpcU were added at either 5 times or 10 times the molar ratio of ExoU (326 pmol or 652 pmol). The same amount of substrate (200 µL) was added, and the rest of the assay was performed as described above. All experiments were performed in triplicate.

### Surface Plasmon Resonance study of the ExoU–SpcU interaction

Binding of ExoU to SpcU and SpcU to ExoU was measured using a Reichert SR7500DC instrument (Reichert Technologies, Depew, NY). SpcU or ExoU (16 µM) in 10 mM sodium acetate buffer pH 6.0 or pH 8.3 was immobilized using the standard amino coupling at 50 µL min^−1^ on a 500,000 Da carboxymethyl dextran hydrogel surface sensor chip (Reichert Technologies, Depew, NY) until saturation was achieved. The running buffer used in all experiments was 10 mM Tris–HCl pH 8.3, 500 mM NaCl, 5 mM 2-Mercaptoethanol. All SPR experiments were performed at 25°C. ExoU (0.8, 1.1, 1.4, 1.7, 2.0 µM) was injected over the SpcU-chip at 40 µL min^−1^ for 3 min to minimize the mass transfer effect. Complete dissociation was achieved after 8 min. Regeneration of the chip was not carried out. Binding was detected as a change in the refractive index at the surface of the chip as measured by response units (RU). A reference flow cell was used to record the background response, and background was subtracted from each sample. *K_d_* values were calculated as ratios of k*_a_*/k*_d_* determined from kinetic experiments. Each experiment included duplicates of each solute concentration. Data models were fit using SCRUBBER-2 available at http://www.biologic.com.au, CLAMP [42] and exported to Origin 8.0 for plotting.

### Isothermal Titration Calorimetry study of the ExoU–SpcU interaction

To characterize thermodynamically the ExoU–SpcU interaction, we conducted isothermal titration calorimetry (ITC) experiments using an iTC_200_ microcalorimeter (MicroCal, Piscataway, NJ). High-concentration stock solutions of ExoU and SpcU were dialyzed over night against 10 mM Tris-HCl pH 8.3 buffer, 200 mM NaCl at 4°C using Slide-A-Lyzer® Dialysis cassettes (Thermo Scientific, Rockford, IL) with 20 kDa and 7 kDa molecular weight cut-off, respectively. For the experiments, the proteins were diluted with the same dialysis buffer to desired concentrations. First, 240 µM (120 µM – SpcU dimer concentration) SpcU was used to titrate 15 µM ExoU. A total 19 injections of SpcU (2 µL each) were done at 22°C. Second, 5 µM (2.5 µM – SpcU dimer concentration) SpcU was titrated with 50 µM ExoU. A total of 15 injections (2.5 µL each) in the same aforementioned buffer and at the same temperature were done. The data sets were fit to a “one set of sites” model to determine the binding stoichiometry (*N*), binding constant (*K*), and thermodynamic parameters (enthalpy and entropy changes) using the Origin software provided by the manufacturer.

### Size exclusion chromatography with multi-angle laser light scattering (SEC-MALS)

Molecular weights of the ExoU–SpcU complex, ExoU and SpcU were determined by conducting SEC-MALS experiments using Agilent Technologies 1100 LC HPLS system (Agilent Technologies, Santa Clara, CA) equipped with Dawn® Heleos™II 18-angle MALS light scattering detector, Optilab® T-rEX™ (refractometer with EXtended range) refractive index detector, WyattQELS™ quasi-elastic (dynamic) light scattering (QELS) detector and ASTRA software (all four from Wyatt Technology Europe GmbH, Dernbach, Germany). A total of 100 µL (2 mg/mL) of the co-expressed/co-purified ExoU–SpcU complex, ExoU, SpcU or ExoU∶SpcU (1∶2.5 and 1∶8 molar ratios) in 10 mM Tris-HCl pH 8.3 with 200 mM/500 mM NaCl were injected and run on a Superdex 200 10/300 GL column (GE Healthcare, Piscataway, NJ) pre-equilibrated with the same buffer, at flow rate of 0.4 mL/min at 22°C. Bovine serum albumin (BSA) (Sigma-Aldrich Corp., St. Louis, MO) was used as a control.

### Crystallization, crystal handling, and data collection

ExoU (7 mg/mL) and SpcU in buffer A were co-crystallized using 2 to 3 molar excess of the chaperone. The proteins were mixed together and incubated on ice for at least 3 hr. Crystallizability of the mixture of proteins was tested by the sitting-drop vapor diffusion technique in 96-well format using available QIAGEN crystallization screens. Crystals were obtained from the Classics Suite screen (QIAGEN Inc., Valencia, CA) condition H6 (CS-H6) at 22°C.

The native data set was collected at the Life Sciences Collaborative Access Team (LS-CAT) 21-ID-F beamline (λ = 0.97872, collection temperature 100 K) at the Advanced Photon Source (APS), Argonne National Laboratory (ANL). The Pt-derivatized CS-H6 crystals were obtained after soaking in 10 mM potassium platinum(II) tetrachloride (K_2_PtCl_4_) for 6 hr. The data collection for the Pt-derivatized crystal was done on the LS-CAT 21-ID-D beamline (λ = 1.06899, collection temperature 100 K). The CS-H6 crystals were also used for hexatantalum tetradecabromide (Ta_6_Br_14_; MiTeGen, LLC, Ithaca, NY) soaking for 48 hr [43]. The data set for the Ta_6_Br_14_-derivatized crystal was collected at the Structural Biology Center beamline ID-19 (λ = 0.97721, collection temperature 100 K) at the APS, ANL. Data sets were processed with *HKL*-2000/*HKL*-3000. Diffraction images for the deposited structure are available at the Center for Structural Genomics of Infectious Diseases (CSGID) web site (http://www.csgid.org/csgid/pages/home).

### Phase determination and model building

The structure was solved by the multiple isomorphous replacement method. The 2.5 Å Pt-derivatized crystal was not perfectly isomorphous with the native one. Therefore, the isomorphous differences were not used for heavy atom search and the 13 heavy atom positions defining the Pt substructure were found using the anomalous signal only. *SHELXD* [44] within *HKL*-3000 [45], [46] was used for this purpose. The sites were subsequently refined with *MLPHARE* [47]. The correction for radiation-induced specific changes was crucial for success of the heavy atom search [48], [49].

The Ta_6_Br_12_^2+^ clusters were localized using an anomalous difference map calculated to 4 Å. Two clusters were manually positioned to cover the one observed elongated peak. The resulting 2×Ta_6_Br_12_^2+^ substructure was refined together with the Pt-sites. For all tantalum and bromide positions isomorphous and anomalous occupancies were refined. The map was solvent flattened with *DM* [50], and the resulting Hendrickson-Lattman coefficients were combined with initial Hendrickson-Lattman (H-L) coefficients produced by *MLPHARE*. For this purpose CCP4's Clipper utility – Combine Phases – was used [51]. The relative weights were 0.2 for *DM* and 0.8 for *MLPHARE* contributions, respectively. As H-L coefficients generated by *DM* included a *MLPHARE* contribution, this operation was equivalent to five-fold attenuation of phase probability associated with solvent-flattening and histogram matching. This operation had low impact on the quality of the maps visually assessed; however, without it the automatic model building by *Buccaneer* [52] was not successful. Model building was performed in two rounds using *Buccaneer*. After an initial 10 cycles of model building, the resulting model was trimmed and used as a starting point in the next round of model building. The trimming was performed by selecting only the residues with assigned sequence and the main chain atomic B-factors below 40 Å^2^. This part of the model was essentially correct when we later compared it to the final model. The *Buccaneer* model building was followed by the removal of incorrectly placed residues with help of the *Coot* [53] program. The structure was refined with *REFMAC* v.5.5 [54], [55]. The final model had the following Ramachandran statistics: 94.8% (residues in most favored regions); 4.7% (residues in additional allowed regions); and 0.5% (residues in generously allowed regions).

### Software and servers used in the present work

All structure figures were prepared with the *PyMOL* program [56]. The PPM server [57] was used to predict possible membrane-embedded residues of ExoU. Superposition of structures was done in *Coot* using Secondary-Structure Matching (SSM) [58] and *DaliLite* Pairwise comparison of protein structures server [59]. Multiple sequence alignment was done by *CLUSTALW* available at http://www.genome.jp/tools/clustalw/ and figures were prepared with *ESPript* 2.2 [60]. *Risler* [61], incorporated into *ESPript* 2.2, was used to calculate the similarity score. SimilarityGlobalScore of 0.7 and SimilarityDiffScore of 0.5 were used. The *VAST* (Vector Alignment Search Tool) algorithm (available at http://www.ncbi.nlm.nih.gov/Structure/VAST/vastsearch.html) was used to predict the domain boundaries of ExoU [62], [63].

### Author information

The atomic coordinates and structure factors are deposited in Protein Data Bank under accession number 3TU3.

## Supporting Information

Figure S1 **SpcU is a class IA/IB chaperone.** (**A**) A stereo-view of the superposed structures of SpcU (red ribbon) with the following class IA chaperones (grey ribbons): SycT from *Yersinia enterocolitica* (PDB code 2BSJ), SycE from *Yersinia pseudotuberculosis* (PDB code 1JYA), SicP from *Salmonella typhimurium* (PDB code 1JYO), SycH from *Yersinia pestis* (PDB code 1TTW), SigE from *Salmonella enterica* (PDB code 1K3S), SrcA from *S. typhimurium* (PDB code 3EPU), ExsC from *Pseudomonas aeruginosa* (PDB code 3KXY), and AvrPphFOrif1 from *Pseudomonas syringae pv. phaseolicola* (PDB code 1S28). The arrow indicates a region of the helix *α*2′ that is absent in the SycT structure. (**B**) A stereogram of the superposed structures of SpcU (red ribbon) with class IB chaperones (grey ribbons) Spa15 from *Shigella flexneri* (PDB code 1RY9) and InvB from *S. typhimurium* (PDB code 2FM8).
(PDF)

Figure S2 **Similarities in effector–chaperone interactions.** The binding of SpcU to ExoU is similar to that of the chaperones ExsC and InvB to their cognate partners. In all panels, monomers of chaperone proteins are shown for clarity. The *β*1-strand of ExoU is highlighted to localize the beta interaction motif of the complexes. The 395–402 peptide of ExoU is shown. (**A**) A stereogram of the superposed structures of the ExoU–SpcU complex (domains of ExoU and SpcU are colored as in **Fig. 1**) and the *P. aeruginosa* ExsC–ExsE (grey and brown, respectively) complex (PDB code 3KXY). (**B**) A stereo view of the structures of the ExoU–SpcU complex and the structure of the *S. typhimurium* InvA–InvB (purple and grey, respectively) complex (PDB code 2FM8).
(PDF)

Figure S3 **Similarities in effector–chaperone interactions.** The binding of SpcU to ExoU is similar to that of the chaperones SicP, and SycE to their cognate partners. In all panels, monomers of chaperone proteins are shown for clarity. The *β*1-strand of ExoU is highlighted to localize the beta interaction motif of the complexes. The 395–402 peptide of ExoU is shown. (**A**) A stereogram of the aligned structures of the ExoU–SpcU complex and the *S. typhimurium* SicP–SptP (grey and dark pink, respectively) complex (PDB code 1JYO). (**B**) A stereo view of the superposed structures of the ExoU–SpcU complex and the *Y. pseudotuberculosis* YopE–SycE (orange and grey, respectively) complex (PDB code 1L2W).
(PDF)

Figure S4 **Comparison of patatin-like PLA_2_ folds.** (**A**) A stereogram of the superposed structures of the PLA_2_ domains of ExoU (red) and human cPLA_2_ (green, PDB code 1CJY). (**B**) The structurally aligned catalytic domains of ExoU (red) and plant patatin PLA_2_ (blue, PDB code 1OXW). Some *β*-strands of the *β*-sheet of the catalytic domain of ExoU are labeled in both panels.
(PDF)

Table S1 **Pairwise structural alignment of SpcU with the chaperones of class IA/IB.** *^a^*See the legend of **Fig. S1** for identification of PDB codes.
(DOC)

Table S2 **Root-mean-square deviation (r.m.s.d.) of the PLA_2_ domain of ExoU and the PLA_2_ domains of plant patatin and human cPLA_2_.**
(DOC)

Table S3 **Mutagenesis sites that diminish cytotoxicity of ExoU.** ^a^ [25]. ^b^ [10]. **^c^** [21]. ^d^ [9]. ^e^ [24]. Domain 1 – the chaperone-binding domain, domain 2 – the PLA_2_ domain, domain 3 and domain 4 are domains of the membrane-localization domain (MLD) region.
(DOC)
