# Supplementary material for: Structure of the Type III Secretion Effector Protein ExoU in Complex with Its Chaperone SpcU
Source: PLoS One. 2012 Nov 14;7(11):e49388. doi: 10.1371/journal.pone.0049388 (PMC3498133; doi:10.1371/journal.pone.0049388)
Supplement: Figure S1 — SpcU is a class IA/IB chaperone. (A) A stereo-view of the superposed structures of SpcU (red ribbon) with the following class IA chaperones (grey ribbons): SycT from Yersinia enterocolitica (PDB code 2BSJ), SycE from Yersinia pseudotuberculosis (PDB code 1JYA), SicP from Salmonella typhimurium (PDB code 1JYO), SycH from Yersinia pestis (PDB code 1TTW), SigE from Salmonella enterica (PDB code 1K3S), SrcA from S. typhimurium (PDB code 3EPU), ExsC from Pseudomonas aeruginosa (PDB code 3KXY), and AvrPphFOrif1 from Pseudomonas syringae pv. phaseolicola (PDB code 1S28). The arrow indicates a region of the helix α2′ that is absent in the SycT structure. (B) A stereogram of the superposed structures of SpcU (red ribbon) with class IB chaperones (grey ribbons) Spa15 from Shigella flexneri (PDB code 1RY9) and InvB from S. typhimurium (PDB code 2FM8). (PDF) [file pone.0049388.s001.pdf]

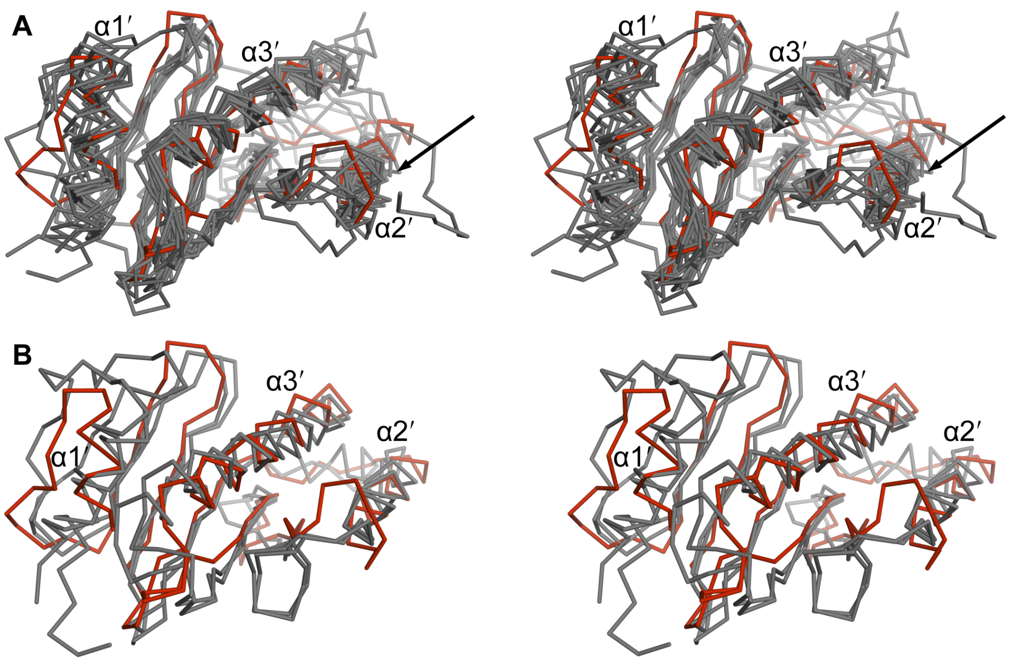

Figure S1. SpcU is a class IA/IB chaperone. (**A**) A stereo-view of the superposed structures of SpcU (red ribbon) with the following class IA chaperones (grey ribbons): SycT from *Yersinia enterocolitica* (PDB code 2BSJ), SycE from *Yersinia pseudotuberculosis* (PDB code 1JYA), SicP from *Salmonella typhimurium* (PDB code 1JYO), SycH from *Yersinia pestis* (PDB code 1TTW), SigE from *Salmonella enterica* (PDB code 1K3S), SrcA from *S. typhimurium* (PDB code 3EPU), ExsC from *Pseudomonas aeruginosa* (PDB code 3KXY), and AvrPphFOrif1 from *Pseudomonas syringae pv. phaseolicola* (PDB code 1S28). The arrow indicates a region of the helix *α*2′ that is absent in the SycT structure. (**B**) A stereogram of the superposed structures of SpcU (red ribbon) with class IB chaperones (grey ribbons) Spa15 from *Shigella flexneri* (PDB code 1RY9) and InvB from *S. typhimurium* (PDB code 2FM8).
