# Supplementary material for: Structure of the Type III Secretion Effector Protein ExoU in Complex with Its Chaperone SpcU
Source: PLoS One. 2012 Nov 14;7(11):e49388. doi: 10.1371/journal.pone.0049388 (PMC3498133; doi:10.1371/journal.pone.0049388)
Supplement: Figure S2 — Similarities in effector–chaperone interactions. The binding of SpcU to ExoU is similar to that of the chaperones ExsC and InvB to their cognate partners. In all panels, monomers of chaperone proteins are shown for clarity. The β1-strand of ExoU is highlighted to localize the beta interaction motif of the complexes. The 395–402 peptide of ExoU is shown. (A) A stereogram of the superposed structures of the ExoU–SpcU complex (domains of ExoU and SpcU are colored as in Fig. 1 ) and the P. aeruginosa ExsC–ExsE (grey and brown, respectively) complex (PDB code 3KXY). (B) A stereo view of the structures of the ExoU–SpcU complex and the structure of the S. typhimurium InvA–InvB (purple and grey, respectively) complex (PDB code 2FM8). (PDF) [file pone.0049388.s002.pdf]

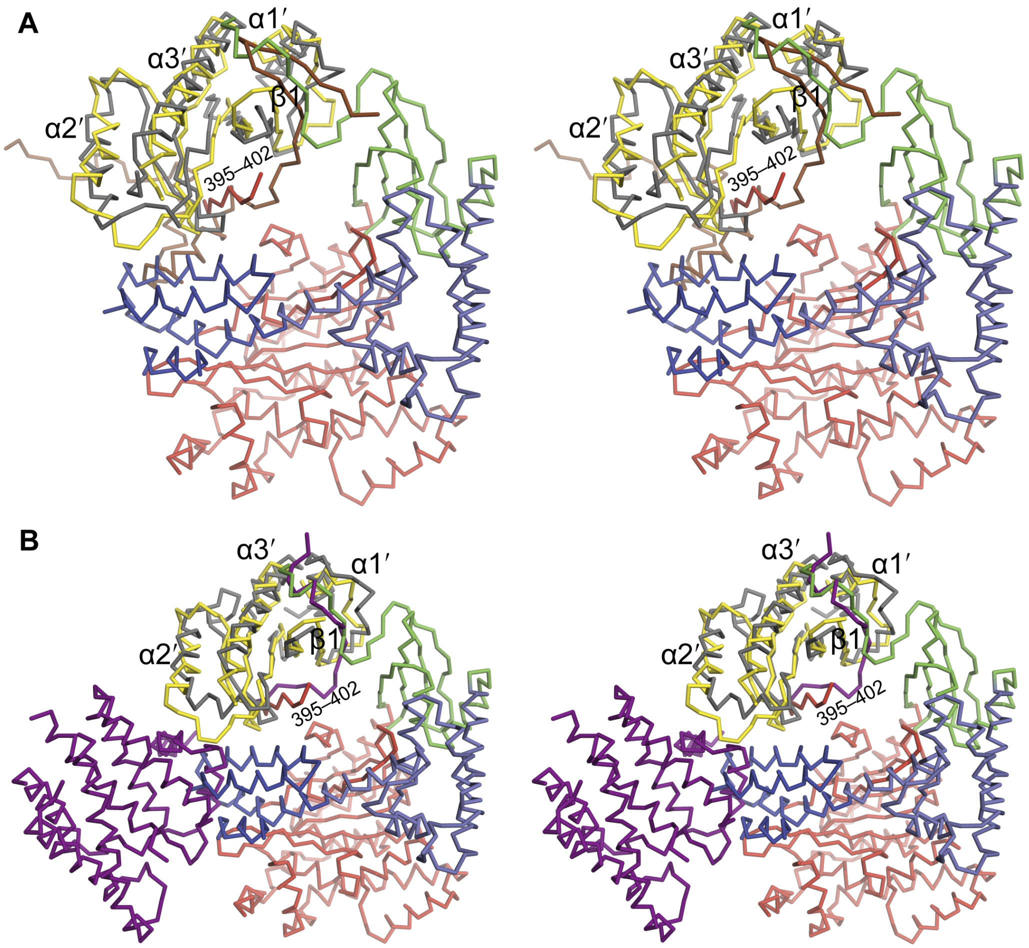

Figure S2. Similarities in effector–chaperone interactions. The binding of SpcU to ExoU is similar to that of the chaperones ExsC and InvB to their cognate partners. In all panels, monomers of chaperone proteins are shown for clarity. The *β*1-strand of ExoU is highlighted to localize the beta interaction motif of the complexes. The 395–402 peptide of ExoU is shown. (**A**) A stereogram of the superposed structures of the ExoU–SpcU complex (domains of ExoU and SpcU are colored as in **Fig. 1**) and the *P. aeruginosa* ExsC–ExsE (grey and brown, respectively) complex (PDB code 3KXY). (**B**) A stereo view of the structures of the ExoU–SpcU complex and the structure of the *S. typhimurium* InvA–InvB (purple and grey, respectively) complex (PDB code 2FM8).
