# Supplementary material for: Structure of the Type III Secretion Effector Protein ExoU in Complex with Its Chaperone SpcU
Source: PLoS One. 2012 Nov 14;7(11):e49388. doi: 10.1371/journal.pone.0049388 (PMC3498133; doi:10.1371/journal.pone.0049388)
Supplement: Figure S3 — Similarities in effector–chaperone interactions. The binding of SpcU to ExoU is similar to that of the chaperones SicP, and SycE to their cognate partners. In all panels, monomers of chaperone proteins are shown for clarity. The β1-strand of ExoU is highlighted to localize the beta interaction motif of the complexes. The 395–402 peptide of ExoU is shown. (A) A stereogram of the aligned structures of the ExoU–SpcU complex and the S. typhimurium SicP–SptP (grey and dark pink, respectively) complex (PDB code 1JYO). (B) A stereo view of the superposed structures of the ExoU–SpcU complex and the Y. pseudotuberculosis YopE–SycE (orange and grey, respectively) complex (PDB code 1L2W). (PDF) [file pone.0049388.s003.pdf]

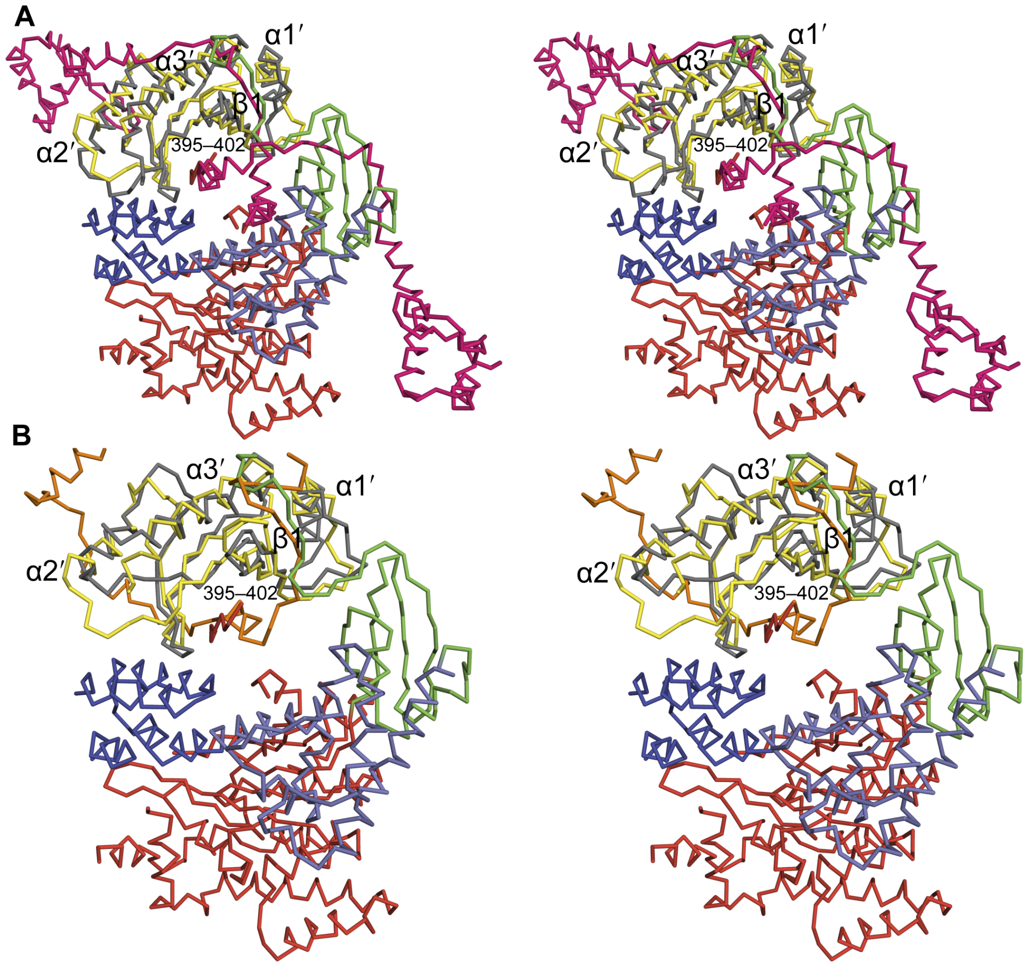

Figure S3. Similarities in effector–chaperone interactions. The binding of SpcU to ExoU is similar to that of the chaperones SicP, and SycE to their cognate partners. In all panels, monomers of chaperone proteins are shown for clarity. The *β*1-strand of ExoU is highlighted to localize the beta interaction motif of the complexes. The 395–402 peptide of ExoU is shown. (**A**) A stereogram of the aligned structures of the ExoU–SpcU complex and the *S. typhimurium* SicP–SptP (grey and dark pink, respectively) complex (PDB code 1JYO). (**B**) A stereo view of the superposed structures of the ExoU–SpcU complex and the *Y. pseudotuberculosis* YopE–SycE (orange and grey, respectively) complex (PDB code 1L2W).
