# Supplementary material for: Structure of the Type III Secretion Effector Protein ExoU in Complex with Its Chaperone SpcU
Source: PLoS One. 2012 Nov 14;7(11):e49388. doi: 10.1371/journal.pone.0049388 (PMC3498133; doi:10.1371/journal.pone.0049388)
Supplement: Figure S4 — Comparison of patatin-like PLA2 folds. (A) A stereogram of the superposed structures of the PLA2 domains of ExoU (red) and human cPLA2 (green, PDB code 1CJY). (B) The structurally aligned catalytic domains of ExoU (red) and plant patatin PLA2 (blue, PDB code 1OXW). Some β-strands of the β-sheet of the catalytic domain of ExoU are labeled in both panels. (PDF) [file pone.0049388.s004.pdf]

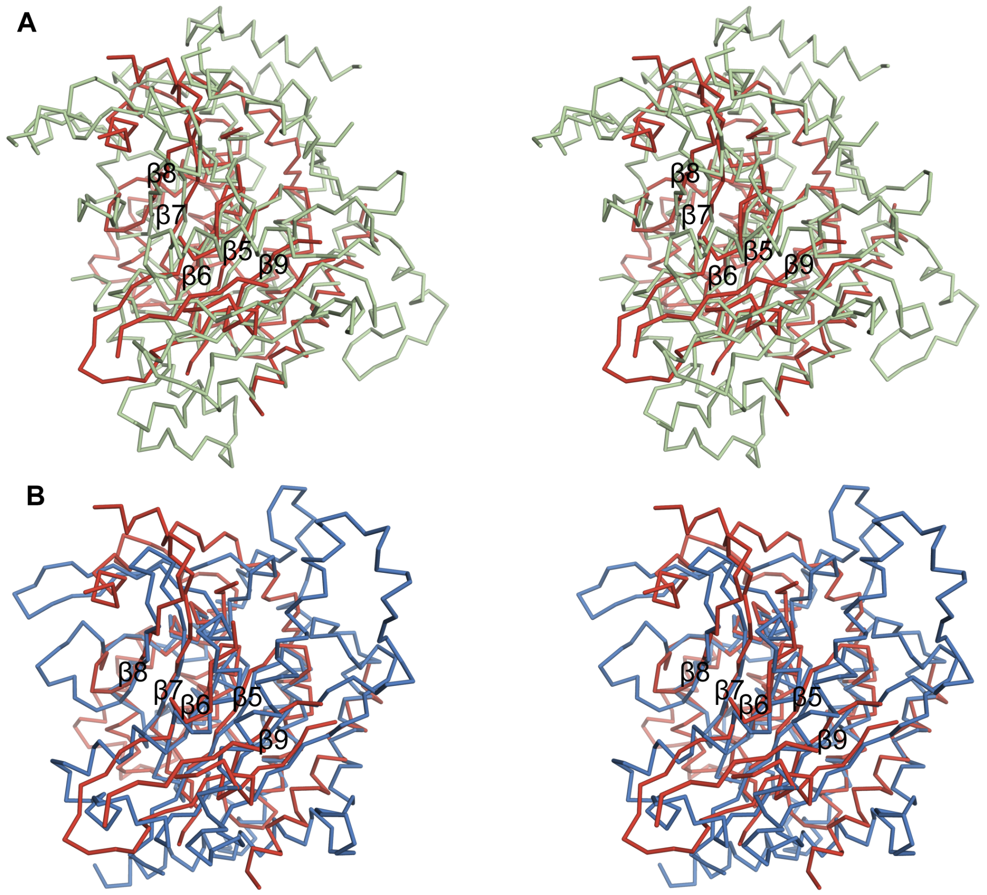


**Figure S4. Comparison of patatin-like $PLA_2$ folds.** (**A**) A stereogram of the superposed structures of the $PLA_2$ domains of ExoU (red) and human $cPLA_2$ (green, PDB code 1CJY). (**B**) The structurally aligned catalytic domains of ExoU (red) and plant patatin $PLA_2$ (blue, PDB code 1OXW). Some *β*-strands of the *β*-sheet of the catalytic domain of ExoU are labeled in both panels.
