# Supplementary material for: Structure of the Type III Secretion Effector Protein ExoU in Complex with Its Chaperone SpcU
Source: PLoS One. 2012 Nov 14;7(11):e49388. doi: 10.1371/journal.pone.0049388 (PMC3498133; doi:10.1371/journal.pone.0049388)
Supplement: Table S1 — Pairwise structural alignment of SpcU with the chaperones of class IA/IB. aSee the legend of Fig. S1 for identification of PDB codes. (DOC) [file pone.0049388.s005.doc]

**Table S1. Pairwise structural alignment of SpcU with the chaperones of class IA/IB.**

| **PDB code*^a^*: chain** | **# of residues**  **in alignment** | **RMSD, Å** | **Sequence identity within aligned region, %** |
| --- | --- | --- | --- |
| 2BSJ: A | 105 | 2.7 | 12 |
| B | 102 | 3.0 | 13 |
| 1JYA: A | 106 | 2.4 | 20 |
| B | 112 | 2.7 | 19 |
| 1JYO: A | 117 | 2.7 | 16 |
| B | 117 | 2.8 | 16 |
| C | 117 | 2.8 | 16 |
| D | 117 | 2.8 | 15 |
| 1TTW: A | 108 | 2.7 | 11 |
| 1K3S: A | 103 | 3.4 | 14 |
| B | 102 | 2.8 | 14 |
| 3EPU: A | 113 | 2.8 | 11 |
| B | 101 | 4.0 | 12 |
| 3KXY: A | 116 | 3.3 | 15 |
| B | 117 | 3.3 | 16 |
| C | 117 | 3.3 | 15 |
| D | 115 | 3.2 | 16 |
| E | 114 | 3.0 | 15 |
| F | 116 | 3.1 | 13 |
| G | 116 | 3.2 | 14 |
| H | 116 | 3.0 | 14 |
| I | 116 | 3.2 | 15 |
| J | 115 | 3.1 | 16 |
| K | 114 | 3.1 | 16 |
| L | 117 | 3.1 | 15 |
| 1S28: A | 106 | 2.5 | 9 |
| B | 106 | 2.5 | 10 |
| C | 106 | 2.6 | 10 |
| D | 108 | 2.7 | 10 |
| 1RY9: A | 110 | 3.0 | 10 |
| B | 112 | 3.0 | 10 |
| C | 111 | 3.0 | 10 |
| D | 112 | 3.0 | 10 |
| 2FM8: A | 113 | 3.2 | 12 |
| B | 104 | 2.8 | 13 |

*^a^*See the legend of Figure S1 for identification of PDB codes.
