# Supplementary material for: Structure of the Type III Secretion Effector Protein ExoU in Complex with Its Chaperone SpcU
Source: PLoS One. 2012 Nov 14;7(11):e49388. doi: 10.1371/journal.pone.0049388 (PMC3498133; doi:10.1371/journal.pone.0049388)
Supplement: Table S2 — Root-mean-square deviation (r.m.s.d.) of the PLA2 domain of ExoU and the PLA2 domains of plant patatin and human cPLA2. (DOC) [file pone.0049388.s006.doc]

**Table S2. Root-mean-square deviation (r.m.s.d.) of the PLA_2_ domain of ExoU and the PLA_2_ domains of plant patatin and human cPLA_2_.**

| **PDB code: chain** | **# of residues**  **in alignment** | **r.m.s.d., Å** | **Sequence identity within aligned region, %** |
| --- | --- | --- | --- |
| 1CJY: A | 183 | 2.92 | 17.5 |
| B | 181 | 2.90 | 16 |
| 1OXW: A | 164 | 2.64 | 15.9 |
| B | 167 | 2.73 | 15.6 |
| C | 167 | 2.74 | 15.6 |
