# Supplementary material for: Structure of the Type III Secretion Effector Protein ExoU in Complex with Its Chaperone SpcU
Source: PLoS One. 2012 Nov 14;7(11):e49388. doi: 10.1371/journal.pone.0049388 (PMC3498133; doi:10.1371/journal.pone.0049388)
Supplement: Table S3 — Mutagenesis sites that diminish cytotoxicity of ExoU. a [25]. b [10]. c [21]. d [9]. e [24]. Domain 1 – the chaperone-binding domain, domain 2 – the PLA2 domain, domain 3 and domain 4 are domains of the membrane-localization domain (MLD) region. (DOC) [file pone.0049388.s007.doc]

**Table S3. Mutagenesis sites that diminish cytotoxicity of ExoU.**

|  | **5-amino-acid linker insertions after the following residues^a^** | **5-amino-acid linker insertions before the following residues^b^** | **Single amino acid substitutions^c^** | **Single amino acid substitutions that decreased cytotoxicity by 50% or more^d^** | **Truncation studies suggest that the following region is necessary for cytotoxicity^e^** |
| --- | --- | --- | --- | --- | --- |
| **Domain 1** |  | A487 |  |  |  |
| **Domain 2** | L109 | V108 | G111 |  |  |
|  | A117 | G113 | G112 |  |  |
|  | D229 | A117 | G113 |  |  |
|  | L458 | S137 | G140 |  |  |
|  | G463 | E224 | S141 |  |  |
|  |  | N283 | S142 |  |  |
|  |  | T285 | A143 |  |  |
|  |  | P294 | G144 |  |  |
|  |  | K325 | G145 |  |  |
|  |  | I349 | G286 |  |  |
|  |  | V351 | D344 |  |  |
|  |  | P352 | G345 |  |  |
|  |  | V353 | G346 |  |  |
|  |  | R461 |  |  |  |
| **Domain 3** |  | T519 |  |  |  |
| **Domain 4** |  | V604 |  | I609 | 679-683 |
|  |  | N608 |  | Q623 |  |
|  |  | Y619 |  | N627 |  |
|  |  |  |  | I654 |  |
|  |  |  |  | R661 |  |
|  |  |  |  | A678 |  |

^a^[23]. ^b^[10]. **^c^**[17]. ^d^[9]. ^e^[22]. Domain 1 – the chaperone-binding domain, domain 2 – the PLA_2_ domain, domain 3 and domain 4 are domains of the membrane-localization domain (MLD) region.
